# Health-related quality of life among adult HIV positive patients: assessing comprehensive themes and interrelated associations

**DOI:** 10.1007/s11136-019-02203-y

**Published:** 2019-05-16

**Authors:** C. den Daas, G. E. L. van den Berk, M. -J. T. Kleene, E. S. de Munnik, J. G. Lijmer, K. Brinkman

**Affiliations:** 1 Centre for Infectious Disease Prevention, National Institute of Public Health and the Environment (RIVM), Bilthoven, The Netherlands; 2 Interdisciplinary Social Science, Utrecht University, Utrecht, The Netherlands; 3 Internal Medicine, OLVG, Amsterdam, The Netherlands; 4 Internal Medicine, Catharina Hospital (Catharina Ziekenhuis Eindhoven, CZE), Eindhoven, The Netherlands; 5 Psychiatry and Medical Psychology, OLVG, Amsterdam, The Netherlands

**Keywords:** HIV, Health-related quality of life, Social stigma, Sexuality, Social support, Value based health care

## Abstract

**Purpose:**

We selected and evaluated a comprehensive set of themes that encompass health-related quality of life (HRQOL) among HIV patients, which enables clinicians to tailor care to individual needs, follow changes over time and quantify returns on health care investments and interventions.

**Methods:**

HIV patients (*N* = 250) of two Dutch HIV clinics were invited to complete an online survey comprised of a set of (adaptations of) validated questionnaires measuring eight themes, including general health (SF-12), stigma (short stigma scale), social support (SSL12-I), self-esteem (SISE), sexuality problems, anxiety and depression (HADS), sleeping difficulties (SCL90-Sleep) and perceived side-effects.

**Results:**

Findings from 170 (response rate 68%) patients (Male = 159, 94.1%) showed that questionnaires had high internal consistency, and most themes significantly correlated (r’s .21 to − .69, *p* < .05) in the expected directions. Exploring cut-off scores shows that a significant proportion of patients score outside of the desired range on single themes (between 16.0 and 73.1%), and many patients on multiple themes simultaneously (8.9% on 5 or more themes). Regression analysis showed that social support, self-esteem and sexuality problems were associated with general health (*R* = .48, *R*^2^ = .23, *F*(4,145) = 10.57, *p *< .001); adding anxiety and depression, sleeping difficulties and perceived side-effects explained 51.2% of the variance in total (*R* = .72, ∆*R*^2^ = .29, *F*(3, 142) = 27.82, *p* < .001).

**Conclusions:**

We succeeded in developing a questionnaire that comprehensively assesses HRQOL. HRQOL of the majority of Dutch HIV patients could be improved. The themes strongly influenced each other, therefore insights into any of the themes could inform interventions to improve HRQOL, and increase attention to these themes in routine consultations between patients and health care professionals.

## Introduction

Hospitals have many sometimes-conflicting goals, such as patient-centredness, safety, quality, cost-effectiveness. To achieve these goals clear aims have to be set, while the overarching goal of health care is to improve patients’ health outcomes. Value Based Health Care (VBHC) aims to improve patient value, by focusing on their health outcomes, instead of process factors. These outcomes are divided by the cost of care, resulting in highest value for patients [1, 2]. To determine value or quality of care, clearly defined measurable outcomes are needed. These outcomes can be divided in three tiers, outcomes related to survival and mortality, outcomes related to recovery and finally outcomes related to sustainability [2]. In VBHC, it is further stated that value should be measured for defined groups with similar needs. One such group is patients living with human immunodeficiency virus (HIV).

In the Netherlands, HIV care is freely accessible in 26 designated hospitals. The OLVG hospital has the largest HIV clinic in the country, and started to organize its HIV care according to the principles of VBHC for quality of care monitoring. In close collaboration with patients several outcome indicators were defined, varying form measurable medical parameters like viral control and cardiovascular risk, but also patient related outcomes like health-related quality of life (HRQOL), both related to sustainability [2]. Assessment of HRQOL can be useful to tailor care, since it enables caregivers to focus on individual needs during consultations. Furthermore, systematic assessment of changes in HRQOL over time can serve to monitor and evaluate interventions and quantify returns on health care investments [3].

HRQOL refers to how health impacts people’s ability to function in daily life and their perceived well-being in physical, mental and social domains of life [4]. Previous studies have already shown that being diagnosed with HIV or AIDS and experiencing disease-related symptoms is associated with lower HRQOL [3, 5–9]. Furthermore, HRQOL is directly associated with clinically relevant outcomes in HIV positive patients, such as treatment adherence and viral suppression [10, 11]. Adherence to HIV treatment and viral suppression are important to improve treatment effectiveness [12–15], prevent development of drug-resistant HIV-strains, and onward transmission of HIV [16–18].

Based on literature, expert opinion and discussions with patients eight themes were identified as important determinants for HRQOL. A previous qualitative study interviewing patients identified themes that were important to patients [19]. These themes were discussed with expert internists, HIV nurses and psychiatrists from the OLVG hospital, whom also indicated that sleeping problems and experienced side-effects should be assessed. Finally, the literature was consulted, and themes that both influenced HRQOL and clinically relevant outcomes among HIV patients were included (Table 1). The comprehensive set of questionnaires therefore included general health (also often used as a HRQOL measure); and several psychological themes, such as stigma, social support, self-esteem and sexuality problems (including both questions on dysfunctions but also satisfaction). In addition, we measured the clinical themes anxiety and depression, sleeping difficulties and perceived side-effects. In previous studies, most of the themes have been investigated separately, in combination with only few other determinants or using just one or two items to assess separate themes. When multiple themes are assessed almost all themes are found to be interrelated. In Table 1, we have provided an overview of established relations between the themes, and effects themes reported to have on clinically relevant HIV outcomes.

**Table 1:** Themes that encompass health-related quality of life among HIV positive patients, and the associations with clinical and psychological outcomes, and medical HIV-related health outcomes, including the expected directions of the associations (positive or negative)

Themes	Clinical and psychological outcomes	HIV-related health outcomes
Quality of life (physical and mental health)	Stigma (−) [54, 62] Social support (+) [51, 62, 63] Physical health [64] Self-esteem (+) [65] Sexuality problems (−) [66] Anxiety and depression (−) [51] Sleeping difficulties (−) [67] Perceived side-effects (−) [51, 68]	Disease severity (−) Physical health [69] Treatment adherence (+) Mental health [64]
Stigma	Quality of life (−) [44, 54, 55] Social support (−) [25, 43, 44, 54, 62, 70] Self-esteem (−) [25, 43, 44, 54] Sexuality problems (+) [66] Anxiety and depression (+) [25, 54, 62, 70, 71] Sleeping difficulties (no association found) Perceived side-effects (+) [54]	Engagement in care (−) [72] Treatment adherence (−) [43, 72] HIV symptoms (+) [70]
Social support	Quality of life (+) [73] Self-esteem (+) [43, 44] Sexuality problems (no association) Anxiety and depression (−) [43, 62, 71] Sleeping difficulties (∓) [74] Perceived side-effects (−) [63]	Indirect effect, offer coping mechanism for other themes that do affect medical HIV outcomes directly [43, 44, 65, 73]
Self-esteem	Quality of life (+) [44] Sexuality problems (−) [75] Anxiety and depression (−) [44] Sleeping difficulties (−) [76] Perceived side-effects (no association)	
Sexuality problems	Quality of life (−) [66, 77] Anxiety and depression (+) [66] Sleeping difficulties (+) [78] Perceived side-effects (+) [66]	Treatment adherence (−) [66, 79]
Anxiety and depression	Quality of life (−) [71, 80, 81] Sleeping difficulties (+) [82] Perceived side-effects (+) [83]	Treatment adherence (−) [84] Treatment outcomes (−), functionality (−), and mortality (+) [71]
Sleeping difficulties	Quality of life (−) [5, 77] Perceived side-effects (+) [79]	Disease severity (+) [77, 79]
Perceived side-effects	Quality of life (−) [79]	Treatment adherence (−) [79, 85]

The plus (+) and minus (−) signs reflect the directions of the associations (positive or negative). For example, Stigma (−) in the first column and row means a negative association between quality of life and stigma, as quality of life increases stigma decreases

*HIV* human immunodeficiency virus

The objective of our study was to explore the psychometric qualities of the questionnaires, the associations between the themes, and HRQOL among HIV patients receiving care in the Netherlands. The comprehensive set of themes that encompass HRQOL among HIV positive patients was evaluated, independently of influencing factors such as gender, sexual orientation and other social demographic variables.

## Methods

### Participants and procedure

Patients visiting the HIV out-patient clinics (OLVG Amsterdam and the Catharina Hospital Eindhoven (CZE)) between 21 April 2016 and 21 May 2016 were asked to participate by the HIV nurse or doctor during their routine consultations. Inclusion criteria were age over 18 years, and proficient in the written Dutch or English language, as the questionnaire was only available in Dutch and English. In total 250 patients agreed to receive an email with an invitational link to the online survey. In the OLVG, HIV nurse provided information and informed consent was signed on side, thereafter the link to the questionnaire was sent. In the CZE, practically receiving all information and signing the informed consent was not possible. Therefore, patients who indicated to be willing to participate received more information through the link, including contact information for questions, subsequently an online informed consent followed. After the informed consent, the questionnaire followed, which took approximately 15 min to complete.

The Dutch Medical Research Involving Human Subjects Act (Dutch acronym: WMO) does not apply for this study, because no particular behaviour is imposed on participants and there were no (medical) interventions other than routine clinical care. A waiver for full medical ethical review was obtained from Medical Research Ethics Committee United (MEC-U).

### Measures

The measures of the eight chosen themes had to fulfil several criteria. The questionnaires had to be (a) shown reliable and valid, preferably previously tested on Dutch HIV patients; (b) reasonably short; (c) suited for patients with HIV; (d) used in previous studies, preferably often and in many contexts and (e) preferably provide (clinically) relevant cut-off points.

*Quality of life* was assessed with the Short-Form-12 Health Survey (SF-12) [20]. Ware et al. [20] argued that the abbreviated SF-12 validly covers HRQOL and the two subscales: physical and mental health. We used the standard recall period of 4 weeks [21]. Scores range from 0 to 100 (reflecting worst to best health). As we do not have norm scores for our population we analysed scores on the 0-100 scale, with a cut-off criterion of 50 [20].

*Anxiety and depression* was measured using the 14 items of the Hospital Anxiety and Depression Scale (HADS), with two subscales seven items each [22]. Scores on the total scale range from 0 to 42; higher scores represent more distress. A cut-off of 15 on the total scale was used to identify people with a potential depressive disorder [23]. The scores on both subscales range from 0 to 21. As suggested by Bjelland and colleagues we employed a cut-off of nine to identify potential clinical cases of anxiety and depression [24].

*Stigma* was assessed with a ten-item scale, which is an abbreviated version of the often used Berger Stigma scale [25, 26]. It contains the following subscales: “Personalized Stigma”, “Disclosure Concerns”, “Negative Self-Image” and “Public Attitudes”. Average scores were reported, higher average scores indicated greater stigma on the total scale and subscales (range 1–5).

*Social support* was investigated with the 12-item short version of the Social Support List—Interaction with good psychometric properties (SSL-12-I) [27–29]. The scores range from 12 to 48, with higher scores indicating more social support. This scale has three subscales, namely “Everyday support”, “Support in problem situations” and “Esteem support”.

The *Sexuality Problems* questions were adapted from the model of Incentive Motivation [30] and the Natsal-SF [31]. Our scale consists of two subscales with five-point answer scales, namely “Sexual functioning and experience” (five items; “Do you have trouble with getting or keeping an erection”). The second subscale was “Sexual feelings” (three items; “Do you feel anxious about having sex, because you are afraid of passing HIV to others”). Average scores were reported, higher average scores indicated greater stigma on the total scale and subscales (range 1–5).

*Self*-*esteem* was measured using the Single-Item Self-Esteem scale (SISE), on a scale from 1 (not very true of me) to 7 (very true of me) [32]. Robins et al. [32] provided support for the construct validity of this measure; the SISE was highly convergent with the Rosenberg Self-esteem Scale [33].

*Sleeping difficulties* were surveyed with the Sleep Difficulties scale from the Dutch version of the Symptom Checklist-90- Revised (SCL-90-R) [34, 35], following the method of Boelen and Lancee [36]. This scale instructs respondents to rate the presence of three symptoms during the preceding week answered on a five-point scale (1 = not at all, 5 = extremely). We assessed the sum score (range 3–15), higher scores reflecting more sleep difficulties. The cut-off we use was 4.5, which is the score of the average Dutch population [34].

*Side-effects* were measured using a single item: “To what extend do you have the experience that the HIV medication gives you side-effects?” answered on a ten-point scale (1 = not at all, 10 = very much). We do not expect severe side-effects, because this would lead to a switch of medication regiment, therefore validated measures, including lengthy lists of symptoms, were not included in our questionnaire [35, 37].

### Data analysis

The questionnaire in the OLVG was programmed in Questmanager [38] to be able to use the outcomes during routine consultations, which guaranteed safety of the patient data. In the CZE the questionnaire was programmed in EasyResearch [39], and data collected anonymously only for the purpose of this study. To examine the psychometric properties of our scales, we calculated the internal consistency of all total scales (Cronbach’s alpha). Subsequently, we performed principal-components analysis with orthogonal Varimax rotation, extracting factors with Eigenvalues of one or higher [40, 41].

We calculated the total score and subscales scores for our patients on each of the themes, investigated correlations. In addition, we assessed scores related to clinical cut-off scores if available. When a clinical cut-off score was lacking, we used quartiles to divide the participants in four groups to determine upper and lower limits to get some insight into the distribution of patients. We explored how many participants score outside of the desired range on the questionnaires (i.e. upper or lower quartile depending on the scale).

To explore relevance of the chosen set of questionnaires, we performed a hierarchical linear regression analysis on the SF-12 scores and the HADS scores, first adding psychological themes; stigma, social support, self-esteem and sexuality problems. Then we added clinical measures (anxiety and depression, sleeping difficulties and side-effects). Finally, in a third step we added gender and location of the HIV clinic where the participants were treated.

## Results

### Participants

In total 170 HIV positive out-patients participated (response rate 68%). Participants were patients of the CZE or OLVG (43 patients, 24.9% and 127 patients, 75.1% respectively), 159 (94.1%) of the participants were men. One person was an HIV negative partner of one of the patients and was excluded from the analysis.

### Psychometric properties of the questionnaires

All questionnaires and subscales had high internal consistency (Table 2), Cronbach’s alpha between .69 (acceptable) and .90 (excellent). Factor analysis extracted the number of subscales that were expected based on the subscales in reported in the literature, and questions loaded on the factors belonging to the expected subscale. In the HADS, we unexpectedly found a third factor, only one item loads strongest on this factor (“I can enjoy a good book or radio or TV programme”). In the stigma questionnaire, the two items of the “Public Attitudes” subscale, loaded on “Personalized stigma” and “Negative self-image”. Consistent with the original HADS and Stigma scale repeating the analysis with forced factor outcomes confirmed the expected HADS and Stigma subscales. The internal consistency and found structures of the questionnaires warrant continuing with assessing the bidirectional association between the scales.

**Table 2:** Mean scores, standard deviations (SDs), Cronbach’s alphas (*α*), cut-off scores (C), and number (#) and percentage (%) of people scoring suboptimal on all quality of life questionnaires of HIV positive patients (*N* = 170) of the Catharina Hospital Eindhoven and OLVG Amsterdam

	Mean	SD	Cronbach’s *α*	Cut-off*	Number suboptimal	%
Quality of life	69.08	17.46	.80	50	26	16.0
Anxiety and depression	9.62	6.99	.90	15	44	26.0
Stigma	2.03	0.53	.83	Q4: 2.4	37	23.7
Social support	30.99	7.55	.93	Q1: 26	51	31.1
Self-esteem	5.00	1.63	–	Q1: 4.2	38	24.5
Sexuality problems	2.40	0.79	.80	Q4: 2.9	38	24.4
Sleeping difficulties	6.66	2.80	.69	4.5	115	73.1
Perceived side-effects	3.54	2.51	–	Q4: 4.0	35	22.7

*HIV* human immunodeficiency virus, *SD* standard deviation

*Based on literature, if not available based on lower (Q1) or upper (Q4) quartile

Almost all scales were correlated, and correlated in the expected directions (Table 3), based on the literature (Table 1). Additionally, the subscales of all questionnaires showed the same pattern of associations as the total scales (data not shown). Only the stigma scale did not correlate significantly with many of the themes, except for the HADS depression subscale (*r* = .16, *p* < .001), and on the sexuality problems questionnaire, the subscale for sexual feelings (*r* = .33, *p* < .001).

**Table 3:** Significant correlations of all quality of life questionnaires of HIV positive patients (*N* = 170) of the Catharina Hospital Eindhoven and OLVG Amsterdam

	1	2	3	4	5	6	7	8
1 Quality of life								
2 Anxiety and depression	− .69							
3 Stigma	–	–						
4 Social Support	.35	− .46	− .35					
5 Self-esteem	.36	− .56	–	.42				
6 Sexuality Problems	− .35	.34	.33	− .34	–.28			
7 Sleeping difficulties	− .37	.44	–	− .28	.33	− .30		
8 Perceived side-effects	− .33	.25	.21	− .25	–	.37	.24	

The minus sign (−) reflects that some of the correlations are negative

*HIV* human immunodeficiency virus, *SD* standard deviation

### Health-related quality of life among HIV positive people

Using the cut-off scores (Table 2), four (2.4%) patients scored within the desired range on all of the questionnaires; whereas 45 (26.6%) patients scored outside of the desired range on just one of the questionnaires, 56 (33.1%) on two of the questionnaires, 33 (19.5%) on three of the questionnaires, 16 (9.5%) on four of the questionnaires, 10 (5.9%) on five of the questionnaires, 3 (1.8%) on six of the questionnaires, 2 (1.2%) on seven of the questionnaires. None of the patients scored outside of the desired range on all of the questionnaires.

SF-12 scores were significantly predicted through a hierarchical linear regression with the psychological themes: Social Support, Self-esteem and Sexuality problems (see Table 4). Sexuality problems and Self-esteem were the strongest predictors (both *p’s *< .01), followed by Social Support (*p* < .03). Notably, Stigma did not predict SF-12 Health scores. The percentage of explained variance was 22.6% (*R* = .48). Adding HADS scores, Sleeping Difficulties and Experienced Side-effects to the regression, with HADS scores (*p* < .001) as strongest predictor followed by Experienced Side-effects (*p* < .05). The percentage of explained variance significantly increased to 51.2% (*R* = .72, ∆*R*^2^ = .29). The ANOVA from the overall regression equation revealed a statistically significant result for both Step 1, *F*(4,145) = 10.57, *p *< .001, and Step 2, *F*(3, 142) = 27.82, *p* < .001. Finally, adding gender and out-patient clinic (CZE or OLVG, *p*’s > .90) to the regression did not increase the explained variance, *F*(2,140) = 0.01, *p *= .992.

**Table 4:** Prediction of SF-12 Health scores and HADS Anxiety and Depression scores from psychological variables, clinical and medical variables and additional variables (*N *= 170, significant beta’s *p* < .05 in bold)

		Regression on SF-12 scores						Regression on HADS scores					
		Betas step 1	*p*	Betas step 2	*p*	Betas step 3	*p*	Betas step 1	*p*	Betas step 2	*p*	Betas step 3	*p*
Step 1	Stigma	.042	.605	.013	.845	.015	.831	− .046	.518	− .015	.832	− .031	.665
	Social support	**.197**	**.024**	.016	.821	.016	.830	**− .246**	**.002**	**− .199**	**.009**	**− .184**	**.015**
	Self-esteem	**.216**	**.009**	− .060	.416	− .058	.449	**− .425**	**.000**	**− .381**	**.000**	**− .405**	**.000**
	Sexuality Problems	**− .239**	**.004**	− .090	.197	− .091	.220	**.152**	**.035**	.077	.296	.066	.391
Step2	HADS			**− .635**	**.000**	**− .633**	**.000**			–	–	–	–
	Sleeping difficulties			− .045	.504	− .046	.511			**.215**	**.003**	**.226**	**.002**
	Side-effects			**− .131**	**.044**	**− .133**	**.046**			.078	.260	.096	.164
Step 3	Gender					.000	1.000					.070	.299
	Location					.008	.901					− .112	.100
R^2^		**.226**		**.512**		.521		**.396**		**.443**		.462	

Bold printed statistics differ significantly (*p *-value < .05)

*HADS* hospital anxiety and depression scale, *SF*-*12* short-form-12 health survey

As the HADS scores were predictive in the predication of the SF12 scores, we performed a similar hierarchical linear regression with HADS scores as outcome. Social Support, Self-esteem and Sexuality problems were also significantly associated with HADS scores (*p*’s < .04), with Self-esteem as most salient variable. These variables explained 39.6% (*R* = .63) of the total variance, *F*(4,145) = 23.81, *p *< .001. Adding Sleeping Difficulties (*p *= .003) and Experienced Side-effects (*p* = .260) to the regression, with Self-esteem, Sleeping difficulties and Social Support scores as most salient variable (*p*’s < .009). The percentage of explained variance significantly increased 44.3% (*R* = .67, ∆*R*^2^ = .05). The ANOVA from the overall regression equation also revealed a statistically significant result for Step 2, *F*(2,143) = 5.95, *p *= .003. Finally, adding gender and out-patient clinic to the regression did not significantly increase the percentage of explained variance (*R*^2^ = .462, *R* = .68, ∆*R*^2^ = .02, *F*(2,141) = 2.54, *p *= .083).

## General discussion

In this study, we introduce a set of questionnaires for the assessment of the HRQOL among HIV patients during routine clinical practice, but that can also be used to evaluate the separate themes that encompass HRQOL. Our findings showed that the majority of our patients did score outside of the desired range on one or more of our themes. These findings are in line with previous studies that already showed that being diagnosed with HIV is associated with lower HRQOL [3, 5–9]. Notably, in the scales with quartiles as cut-off approximately 25% of the participants scored outside of the desired range, therefore this might exclude people who are experience mild but still clinically relevant issues.

Our questionnaires showed excellent psychometric properties; including the measure we adapted ourselves for sexuality problems. We also found that practically all themes were associated with each other in the directions expected by the literature (Table 1). Furthermore, stigma, social support, self-esteem as well as sexuality problems were associated with HRQOL in a regression analysis. This is important, even though both social support and self-esteem are not directly linked to clinically relevant HIV health outcomes. Social support has been shown to reduce stress experienced because of suffering from long-term illness in general [42], and living with HIV in particular [43]. When people experience some stress, simply being involved in a network, having people around, and feeling part of a group can serve as a buffer against stress and positively affect HRQOL. Similarly, self-esteem, which does influence some of the other themes, could also function as a coping mechanism and positively affect HRQOL in HIV patients [44]. Through these direct and indirect routes, social support and self-esteem could influence clinically relevant outcomes.

Adding anxiety and depression, sleeping difficulties and perceived side-effects in the regression obscured the associations between the psychological themes and HRQOL. Social support and self-esteem were also related to anxiety and depression scores, even after adding sleeping difficulties and perceived side-effects. These findings underscore the importance of systematically measuring themes, in order not to miss the potentially important role of stigma, social support and self-esteem at the expense of the strong association of anxiety and depression with HRQOL scores.

The separate questionnaires in our survey have been used in other populations, making some comparisons possible qualifying the condition of Dutch HIV patients compared to HIV patient in other countries or other subpopulations. Regarding scores on the HADS, these patients experienced less anxiety and depression compared to HIV positive patients from Canada [45]. A study showed that the general Dutch population (18–65 years) scored lower on anxiety and depression, but Dutch general medical patients and psychiatric out-patients scored higher than our HIV positive patients [46]. Furthermore, these patients experienced less stigma compared to 212 young (16–29 years) people living with HIV in the United States [47]. These patients also experienced more social support compared to Dutch elderly [29] or Dutch people, who had a stroke 3 years ago [48]. In contrast, our HIV patients experienced less social support than Dutch patients with spinal cord injury [49]. In addition, these patients were higher in self-esteem than undergraduate students of an American University [32]. In a study that looked at self-esteem across different ages, our HIV patients also scored higher than the highest score age group (60–69 years) [50]. Finally, these participants had more sleeping difficulties compared to the Dutch general population, but less than people, who suffered from prolonged grieving disorder before treatment [34, 36]. A review has shown that HRQOL is influenced by several determinants, and studies measuring HRQOL usually do not include all and the same determinants [51]. Therefore, it is important to assess multiple themes together when evaluating HRQOL in routine clinical care.

Currently, even though the regressions and correlations indicate the relevance of the themes for our HIV patients, we have not yet linked the scores on the questionnaires to other outcome parameters, such as therapy effectiveness, cardiovascular risk and therapy toxicity, nor did we correlate it with patient characteristics. An important next step will be to monitor the themes, their trends over time, and to predict or even influence medical outcomes by offering successful interventions to improve HRQOL.

## Strengths and limitations

A strength of this study is that it is a first step to comprehensively measure HRQOL among HIV patients in the Netherlands. Currently, possible undiagnosed cases of depression are already identified and referred for further psychiatric treatment. However, for several questionnaires clinically relevant cut-off values need to be determined to guide potential interventions. Moreover, for many themes proven effective interventions are not yet available and need to be developed.

A second strength is that the response rate in our study [52, 53] compared to other studies with online survey our response rate is high, even though some of the topics could be perceived as sensitive. This illustrates that participants probably view HRQOL as important, and therefore had a high willingness to participate.

In this first examination, a relatively small number of people participated, and we wanted to keep the request to patients minimal, therefore we have not assessed extensive demographical characteristic and have not requested consent to link this data to the electronic patient files. Consequently, we have not stratified for baseline characteristics, such as gender, age, migration background, HIV history and concurrent co-morbidities. In the near future, we will evaluate the value of these questionnaires in a larger population, where we can analyse the influence of these factors.

Literature shows that stigma is strongly associated with HRQOL in people living with HIV [54–56]. Studies have also shown that stigma is related to depression in India [57], South-Africa [58] and the United States [59]. Some of these studies are focusing on severe stigma, including denial of HIV care, evictions from homes and termination of employment [57, 58]. In our study we could not demonstrate an association between stigma and either HRQOL or anxiety and depression scores. Although, people living with HIV in Netherlands do experience stigma, it appears less severe what might explain the reduced impact on anxiety and depression. It is suggested that the association between stigma and depression might be driven through disclosure avoidance (and resulting lack of social support) [60]. Indeed, we found that social support was associated with HRQOL and anxiety and depression. Nevertheless, other factors we have not assessed might also play a role, such as the amount of time people have been living with HIV (time reduced both stigma and depression) [61]. Finally, we might have to focus on the subscales of stigma to get a clearer view of the association between stigma, HRQOL and anxiety and depression. Negative self-image and public attitudes might influence HRQOL directly, whereas personalized stigma and disclosure concerns might indirectly affect HRQOL through for instance social support [43, 62]. HIV stigma is a multidimensional complex theme, therefore, future research should determine if using this abbreviated scale [26] among Dutch people living with HIV is warranted.

## Conclusion

We succeeded in developing a questionnaire that comprehensively assesses HRQOL among HIV positive patients during routine clinical practice. Using this questionnaire showed that the HRQOL among Dutch HIV patients could be improved. Insights into these themes could inform interventions to improve HRQOL, and at least increase attention to these themes in routine consultations between patients and health care professionals. Measuring HRQOL could thus serve as an intervention in itself, which is in the tradition of VBHC [2]. Currently, this survey is administered yearly among all HIV out-patients in care at the OLVG, to be able to discuss relevant outcomes with patients in their routine consultations; other hospitals are also starting to measure HRQOL using the same themes and questionnaires. In time, we aim to include all hospitals in the Netherlands (and maybe beyond) to provide high quality, identical, but patient-centred HIV care, to all people living with HIV.
